# Proliferative activity of antigen-specific CD154+ T cells against bacterial and fungal respiratory pathogens in cystic fibrosis decreases after initiation of highly effective CFTR modulator therapy

**DOI:** 10.3389/fphar.2023.1180826

**Published:** 2023-06-20

**Authors:** Patience N. Eschenhagen, Petra Bacher, Claudia Grehn, Jochen G. Mainz, Alexander Scheffold, Carsten Schwarz

**Affiliations:** ^1^ Cystic Fibrosis Section, Klinikum Westbrandenburg, Campus Potsdam, Potsdam, Germany; ^2^ HMU Health and Medical University, Potsdam, Germany; ^3^ Department of Pediatric Pneumology, Immunology and Intensive Care Medicine, Charité Universitätsmedizin Berlin, Berlin, Germany; ^4^ Institute of Clinical Molecular Biology, Christian-Albrecht-University of Kiel, Kiel, Germany; ^5^ Institute of Immunology, Christian-Albrecht-University of Kiel and UKSH Schleswig-Holstein, Kiel, Germany; ^6^ Berlin Institute of Health at Charité Universitätsmedizin, Berlin, Germany; ^7^ Cystic Fibrosis Center, Brandenburg Medical School (MHB) University, Brandenburg, Germany; ^8^ Faculty of Health Sciences Joint Faculty of the Brandenburg University of Technology Cottbus-Senftenberg, The Brandenburg Medical School Theodor Fontane and the University of Potsdam, Potsdam, Germany

**Keywords:** cystic fibrosis, antigen-specific T cells, Ki-67, *Pseudomonas aeruginosa*, Aspergillus fumigatus, total IgE, total IgG, highly effective modulator therapy

## Abstract

**Background:** Together with impaired mucociliary clearance, lung disease in cystic fibrosis (CF) is driven by dysregulation of innate and adaptive immunity caused by dysfunctional CFTR (Cystic Fibrosis Transmembrane Conductance Regulator), leading to airway infection and hyperinflamma-tion. The highly effective CFTR modulator therapy (HEMT) elexacaftor/tezacaftor/ivacaftor (ETI) generates substantial improvements in clinical outcomes of people with CF (pwCF) by restoration of CFTR activity. Aberrant immune responses of lymphocytes due to CFTR dysfunction has been described in the past, but not the effects of CFTR restoration by HEMT on these cells. We aimed to examine the effect of ETI on the proliferative activity of antigen-specific CD154 (+) T cells against bacterial and fungal species relevant in CF and on total IgG and IgE as markers of B cell adaptive immunity.

**Methods:** We performed *ex vivo* analyses of Ki-67 expression in antigen-specific CD154 (+) T cells against *Pseudomonas aeruginosa, Staphylococcus aureus, Aspergillus fumigatus, Scedosporium apiospermum* and *Candida albicans* from 21 pwCF by cytometric assay based on antigen-reactive T cell enrichment (ARTE), and analysis of total serum IgE and IgG before and after initiation of ETI.

**Results:** Mean Ki-67 expression in antigen-specific CD154 (+) T cells against *P. aeruginosa, A. fumigatus, S. apiospermum* and *C. albicans*, but not *S. aureus*, mean total serum IgG and mean total serum IgE decreased significantly after initiation of ETI. No correlation was found to change in sputum microbiology of the examined pathogens. Mean BMI and FEV1 increased significantly.

**Conclusion:** HEMT is associated with decreased antigen-specific CD154 (+) T cell proliferation activity in our cohort, independent of findings in sputum microbiology of the examined pathogens. Together with the observed clinical improvement and the decrease in total IgE and IgG, this indicates effects due to CFTR restoration on CD154 (+) T cells by ETI and a reduction of B cell activation with subsequent lower immunoglobulin synthesis under HEMT therapy. These results endorse earlier evidence of CFTR dysfunction in T and B cells leading directly to aberrant immune responses with hyperinflammation.

## 1 Introduction

Knowledge of CFTR (cystic fibrosis transmembrane regulator) dysfunction on epithelial cells and genetic mechanisms in cystic fibrosis (CF) has led to the development of potent symptomatic and mutation-specific drugs. Symptomatic treatment includes effective secretolytic substances and inhalative antibiotics. During the last decade, highly effective CFTR-modulators (HEMT) which restore CFTR activity >25% of wildtype activity have been developed and approved (Zhang et al, 2009). Today, nearly 90% of people with cystic fibrosis (pwCF) have mutations eligible for either CFTR modulator monotherapy or combination therapy (Joynt et al, 2022). Initiation of the highly efficient CFTR modulator therapy (HEMT) elexacaftor/tezacaftor/ivacaftor (ETI), consisting of two CFTR molecule correctors and one CFTR potentiator, resulted in substantial improvements in clinical outcomes of pwCF(3–5).

CF lung disease is also known to be driven by abnormal innate and adaptive immune regulation leading to hyperinflammation and ineffective immune response to respiratory pathogens. Immune dysregulation in CF is both due to genetic and acquired factors. The direct impact of CFTR dysfunction on immune cells is increasingly recognized and under investigation. In neutrophils, defective CFTR causes reduced chlorination resulting in impaired phagocytosis (Painter et al, 2006; Dickerhof et al, 2019; Meoli et al, 2022). In B cells, CFTR has been shown to play a direct role in activation, proliferation and production of inflammatory cytokines, with a subsequent increase in IgG (+)-B cells (Polverino et al, 2019). Already in the 1980ies, progression of cystic fibrosis lung disease could be linked to hypergammaglobulinemia (Wheeler et al, 1984). CFTR-dependent altered regulation of T-cell cytokine secretion leads to a shift towards a proinflammatory state and a predominant Th2 response with exaggerated IgE levels, a predisposition for allergic bronchopulmonary aspergillosis (ABPA) and simultaneously reduced defense against *Pseudomonas aeruginosa* (Moss et al, 1996; Muller et al, 2006; Mueller et al, 2011; Ratner and Mueller, 2012; Duan et al, 2021). The defective Th2 response also drives the development of multispecies cross-reactive Th2-cells as a potential risk factor for ABPA (Schwarz et al, 2023). Heterozygous asthmatic individuals with ABPA are more likely to be carriers of a CFTR mutation (Gamaletsou et al, 2018). *Candida albicans* could be identified by our study group as major inducer of cross-reactive CD154 (+) T cells contributing to *Aspergillus fumigatus*-driven inflammation in the lung in pwCF (Bacher et al, 2019).

So far, the findings have not yet been sufficient to enable the development of targeted antiinflammatory therapies for pwCF. Increasing evidence shows that there may be interindividual differences in the efficacy of HEMT and that HEMT may not be equally effective in different organs and cells in the same individual. This could apply especially to immune cells, as large interindividual differences in immune phenotypes are known to exist in humans (Okada et al, 2021). Initiation of HEMT provides the opportunity to examine in real life the relevance of CFTR-dysfunction found on immune cells *in vitro*, and of changes in host-pathogen interactions in CF. Recently, a partial restoration of macrophage phagocytosis and neutrophil efferocytosis by ETI was observed, and non-responders in the CF group were identified (Zhang et al, 2022). We therefore aimed to investigate the effects of HEMT on the proliferation activity of CD154 (+) T cells with antigenic specificity for relevant respiratory pathogens and of total IgG and IgE in pwCF. Ki-67 antigen was first described in 1983 as a marker present in proliferating cells and is widely used in tumor cell kinetics assessment, but has so far never been used to describe proliferative activity of T cells in CF (Gerdes et al, 1983; Scholzen and Gerdes, 2000).

## 2 Materials and methods

### 2.1 Patient cohort and study design

A total of 21 pwCF were included in the study. All patients gave written consent to participate. Serial samples for antigen-specific CD154 (+) T cells were taken at routine or acute visits or during hospitalization between 6.39 years and 7 days prior to and between 25 days and 2.5 years after initiation of ETI. The mean number of visits per patient was 4.83 (Wheeler et al, 1984; Painter et al, 2006; Dickerhof et al, 2019; Heijerman et al, 2019; Middleton et al, 2019; Polverino et al, 2019; Zemanick et al, 2021; Joynt et al, 2022; Meoli et al, 2022). For Ki-67 analysis, complete data were available from 21 patients for *A. fumigatus*, 14 patients for *S. apiospermum* and *C. albicans*, 8 patients for *S. aureus* and 9 patients for *P. aeruginosa*. In both the pre and post ETI group, two patients were excluded from IgE analysis due to acute ABPA, and due to incomplete data prior to initiation of ETI, 3 patients were excluded from exacerbation analysis. Baseline and demographical data are shown in Table 1.

**TABLE 1:** Demographic characteristics before and after ETI initiation.

Table 1 Patient characteristics	Patients (*n* = 21)
Gender: female/male	62%/38%
Mean age at visit (years) ± SD (range)	28.9 ± 9.4 (8–56)
Genotype	
F508del homozygous	*n* = 10 (47.6%)
F508del heterozygous	*n* = 11 (52.4%)
Exocrine Pancreatic Insufficiency	*n* = 19 (90.5%)
CF-related Diabetes mellitus	*n* = 8 (38.1%)
BMI kg/m^2^ (mean, ±SD (range))	19.4 ± 2.2 (15.4–22.4)
ppFEV1 pre ETI (mean ± SD (range))	43.8 ± 16.8 (23.2–82.0)
Organ Transplantation	*n* = 0
Pulmonary exacerbations per year, 2 years pre ETI (*n* = 18^a^, mean, ±SD (range))	3.3 ± 1.7 (1–5.5)
Pulmonary exacerbations per year, 2 years post ETI (*n* = 18^a^, mean, ±SD (range))	1.5 ± 1.4 (0–3.5), *p* < 0.001
Mean number of visits per patient ±SD (range)	4.83 ± 2.24 (2–10)
Total number of visits pre ETI	*n* = 67
Total number of visits post ETI	*n* = 31
∑ pre + post ETI	*n* = 98
Systemic steroid therapy pre ETI, visits	*n* = 22 (32.8% of pre ETI)
Systemic steroid therapy post ETI, visits	*n* = 9 (29.0% of post ETI), ns^b^
Antifungal therapy pre ETI, visits	*n* = 17 (25.3% of pre ETI)
Antifungal therapy post ETI, visits	*n* = 2 (6.4% of post ETI), *p* < 0.05
ABPA acute + untreated pre ETI, mean total IgE (kU/L) ± SD (range)	*n* = 4, 1630 (1073–2,943)
ABPA acute + untreated post ETI, mean total IgE (kU/L) ± SD (range)	*n* = 4, 3132 (2,327–4,599)
Sputum microbiology (pre ETI, number of visits)	Positive sputum result pre ETI
*Pseudomonas aeruginosa* ^c^ (*n* = 43)	*n* = 33 (82.5%)
*Staphylococcus aureus* ^c^ (*n* = 38)	*n* = 10 (26.3%)
*Aspergillus fumigatus* ^c^ (*n* = 70)	*n* = 24 (32.9%)
*Scedosporium apiospermum/*spp.^c^ (*n* = 53)	*n* = 7 (13.2%)
*Candida albicans* ^c^ (*n* = 53)	*n* = 19 (35.8%)
Other bacteria (*n* = 70)	*n* = 13 (13.3%)
*Achromobacter xylosoxidans*	*n* = 2 (2.8%)
*Burkholderia multivorans*	*n* = 8 (11.4%)
*Serratia marcescens*	*n* = 3 (4.3%)
*Proteus mirabilis*	*n* = 1 (1.4%)
*M. intracellulare-complex*	*n* = 2 (2.8%)
Other fungi^5^ (*n* = 70)	*n* = 12 (17.1%)
*Aspergillus flavus*	*n* = 1 (1.4%)
*Exophiala dermatitidis*	*n* = 7 (10.0%)
*Candida dubliniensis*	*n* = 14 (20.0%)
*Candida guilliermondii*	*n* = 4 (5.7%)
*Candida glabrata*	*n* = 11 (15.7%)
*Penicillium* spp.	*n* = 1 (1.4%)

a Patients with exacerbation data for the respective time frame.

b ns: not significant.

c Patients with Ki-67, analysis available for the respective pathogen.

### 2.2 Data collection and statistical analysis

Sample collection was conducted between May 2014 and December 2022 at Christiane Herzog Cystic Fibrosis Center, Charité Universitätsmedizin Berlin, Germany, and at Cystic Fibrosis Center Westbrandenburg, Campus Potsdam, Health and Medical University Potsdam.

Ethical aspects were considered and approval for the study was gained by the Ethics Committee of Charité Universitätsmedizin Berlin, Germany (No. EA2/121/16) and by the Medical Ethics Committee Brandenburg [Landesärztekammer Brandenburg, No. AS48 (bB)/2021].

Baseline data were collected using patient records and the German patient registry software “Muko.web”. For the analysis of Ki-67, subgroups with all patients with available antigen-specific CD154 (+) T cells before and after initiation of ETI were formed. Microbiology data was analyzed with respect to the subgroups.

Distribution of data was assessed with Shapiro-Wilk test for normal distribution. Associations between binary variables were assessed using Barnard`s exact test, and between quantitative variables using *t*-test. Due to unknown effect sizes, *a priori* power analyses were not performed. Data analyses were performed using Microsoft Excel Version 2301 (Build 16.0.16026.20196) and R version 4.2.1.

### 2.3 Antigen-specific T cells, microbiology and immunoglobulins

Antigen specific CD154 (+) T cells were derived by Antigen-reactive T cell enrichment (ARTE) which allows the detection of antigen reactive T cells against single antigens, as described elsewhere (Bacher et al, 2013). Cells were stained for multiparametric flow cytometry with Ki-67-antibodies (Miltenyi Biotec Cat# 130-119-356, RRID:AB_2857452) and flow cytometry analysis of cytokine-secreting antigen-specific T cells were performed. Antigens for T cell stimulation were chosen due to the relevance and frequency of the respective pathogens in CF, and due to their availability. Except for *C. albicans*, the chosen pathogens are considered to have a major impact on CF lung disease (Bell et al, 2020). However, *C. albicans* is isolated frequently in airways of pwCF, and its influence in CF is unclear (Chotirmall et al, 2010).

Microbiology was collected at every visit spontaneously, as induced sputum (after inhalation of 6% saline) or as physiotherapist assisted induced sputum. Additional microbiological results from other routine visits were analyzed where available if sputum could be expectorated to better reflect colonization status of the patients. Cough swab results were excluded from analysis. Serum was collected from all patients at every visit. Total serum IgG (immunoturbidimetry/nephelometry) and total serum IgE (electrochemiluminescence immunoassay) were determined in our routine laboratories (Labor Berlin/Labor Potsdam) in parallel to Ki-67 analysis.

## 3 Results

### 3.1 Baseline data/demographical data

Statistical analysis reveals a severe phenotype in our cohort, with worse lung function and nutritional status compared to the general CF population in the latest German CF registry data from 2021 (Nährlich et al, 2021). With a mean age of 28.9 years (registry: 23.0 years), the patients were older. The percentage of F508del homozygous patients was similar (47.6% in our cohort vs. 46.6% in the registry), while mean BMI (19.4 kg/m^2^) and mean ppFEV1 (43.8) were lower. Detection rate of *S. aureus* was lower (19.4% vs. 50.0%) and of *P. aeruginosa* higher (64.3% vs. 33.3%) than in all birth cohorts of the German registry population. The rates of pancreatic insufficiency and diabetes were similar to the registry patients. No patients after organ transplantation were included, and the percentages of visits with systemic steroids were similar before and after ETI initiation (32.8 vs. 29.0%, ns). Significantly more pulmonary exacerbations were experienced, and more patients were under antifungal therapy before ETI initiation, mostly receiving long-term antifungal therapy against ABPA. Due to acute ABPA, four patients prior to and four patients after ETI initiation were excluded from total IgE analysis and there was a balanced relation regarding Ki-67 and analysis of *A. fumigatus* specific CD154 (+) cells, total IgG and microbiology. Patient characteristics are listed in Table 1.

### 3.2 General outcome data and microbiology pre and post ETI

A significant increase in mean BMI of 4.0% (19.4 vs. 20.6 kg/m^2^, *p* < 0.01) and mean absolute ppFEV1 of 6.5 (43.8 vs. 50.3, *p* < 0.01) was shown after ETI initiation. We observed a significantly lower pulmonary exacerbation rate in the 2 years past vs. the 2 years prior to ETI initiation (3.3 vs. 1.5, *p* < 0.001). These data confirm the benefits of HEMT in a cohort with a relatively severe mean phenotype in real life. Nevertheless, mean BMI and ppFEV1 after initiation of ETI were still lower than in the German registry population before approval of ETI. In microbiology, we found a significant decrease in *A. fumigatus* sputum detection in our cohort (48.9 vs. 2.0%, *p* < 0.001). For *P. aeruginosa*, *Staphylococcus aureus*, *Scedosporium apiospermum* and *C. albicans* we found no significant changes in sputum detection rate for the respective groups. For general and microbiology outcome data please see Table 2.

**TABLE 2:** Outcome data before and after initiation of ETI.

Table 2 Outcome data pre and post ETI	Patients (*n* = 21)	
BMI kg/m^2^) pre ETI (mean, ±SD (range))	19.4 ± 2.2 (15.4–22.4)	*p* < 0.01**
BMI (kg/m^2^) post ETI (mean ± SD (range))	20.6 ± 2.6 (14.3–24.8)	
ppFEV1 pre ETI (mean ± SD (range))	43.8 ± 16.8 (23.2–82.0)	*p* < 0.01**
ppFEV1 post ETI (mean ± SD (range))	50.3 ± 15.0 (32.0–89.0)	
Pulmonary Exacerbations pre ETI, visits	n = 52 (76.4% of pre ETI)	*p* < 0.05*
Pulmonary Exacerbations post ETI, visits	n = 16 (53.3% of post ETI)	
Total IgE (IU/mL) pre ETI (Non-ABPA, mean ± SD (range))	91.2 ± 98.6 (2.5–326.0)	*p* < 0.01**
Total IgE (IU/mL) post ETI (Non-ABPA, mean ± SD (range))	28.8 ± 22.5 (3.2–78.2)	
Total IgG (g/L) pre ETI (mean ± SD (range))	15.0 ± 3.9 (9.7–23.5)	*p* < 0.001***
Total IgG (g/L) post ETI (mean ± SD (range))	12.3 ± 3.2 (6.5–22.3)	
Sputum microbiology^a^		
*Pseudomonas aeruginosa* pre ETI, visits	*n* = 40 positive: 33 (82.5%)	ns
*Pseudomonas aeruginosa* post ETI, visits	*n* = 38 positive: 27 (71.1%)	
*Staphylococcus aureus* pre ETI, visits	*n* = 38 positive: 10 (26.3%)	ns
*Staphylococcus aureus* post ETI, visits	*n* = 36 positive: 4 (11.1%)	
*Aspergillus fumigatus* pre ETI, visits	*n* = 70 positive: 23 (32.9%)	*p* < 0.001**
*Aspergillus fumigatus* post ETI, visits	*n* = 53, positive: 1 (1.9%)	
*Scedosporium apiospermum* pre ETI, visits	*n* = 53, positive: 7 (13.2%)	ns
*Scedosporium apiospermum* post ETI, visits	*n* = 45, positive: 5 (11.1%)	
*Candida albicans* pre ETI, visits	*n* = 53, positive: 19 (35.8%)	ns
*Candida albicans* post ETI, visits	*n* = 45, positive: 21 (46.7%)	

a At visits with Ki-67, analysis available for the respective pathogen.

### 3.3 Total IgE and total IgG

**FIGURE 1:**
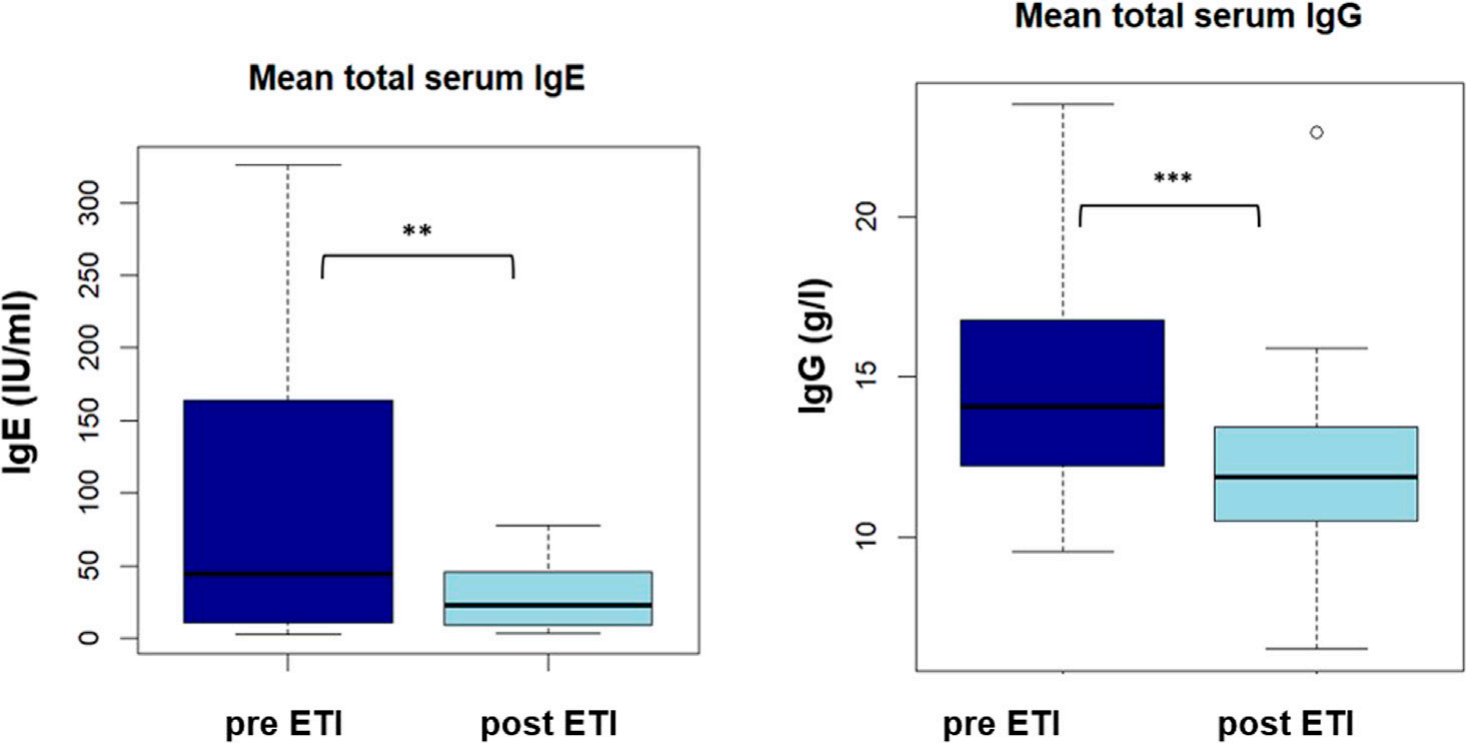
Boxplot showing mean total serum lgE and lgG (IU/ml) pre and post ETI **: *p* < 0,01.

Mean total serum IgE and mean total serum IgG decreased significantly after ETI initiation (91.2 vs. 28.8 IU/mL, *p* < 0.01 and 15,0 vs. 12.3 g/L, *p* < 0.001, please see Figure 1A + B).

### 3.4 Ki-67 expression of antigen-specific CD154 (+)-T cells

**FIGURE 2:**
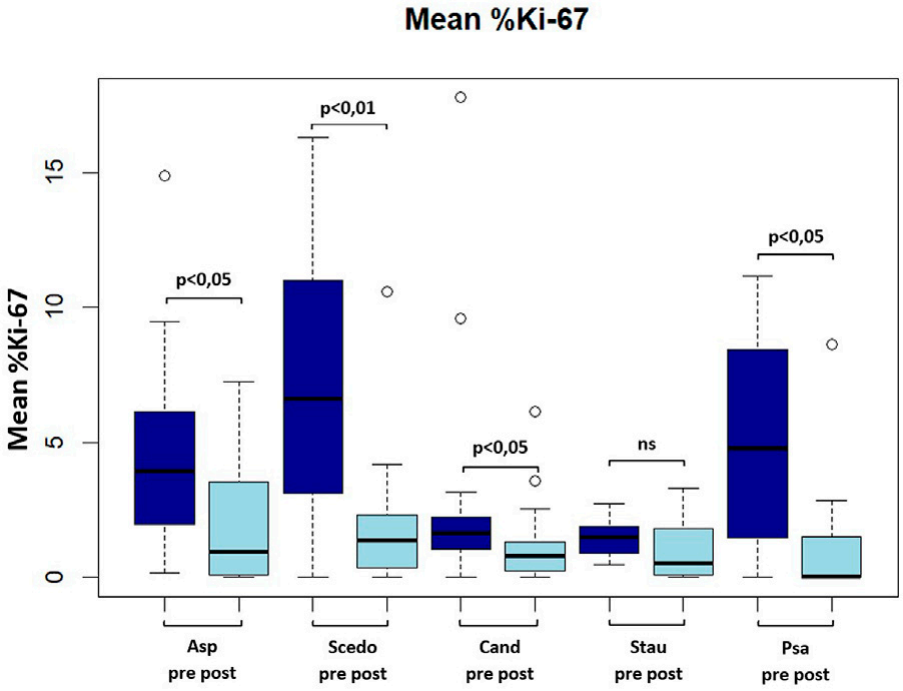
Boxplot showing mean frequency (in percent) of Ki-67(+) antigen-specific CD154(+) Tcells pre and post ETI. Asp, aspergillus fumigatus; Scedo, scedosporium apiospermum; Cand, candida albicans; Stau, staphylococcus aureus.

**FIGURE 3:**
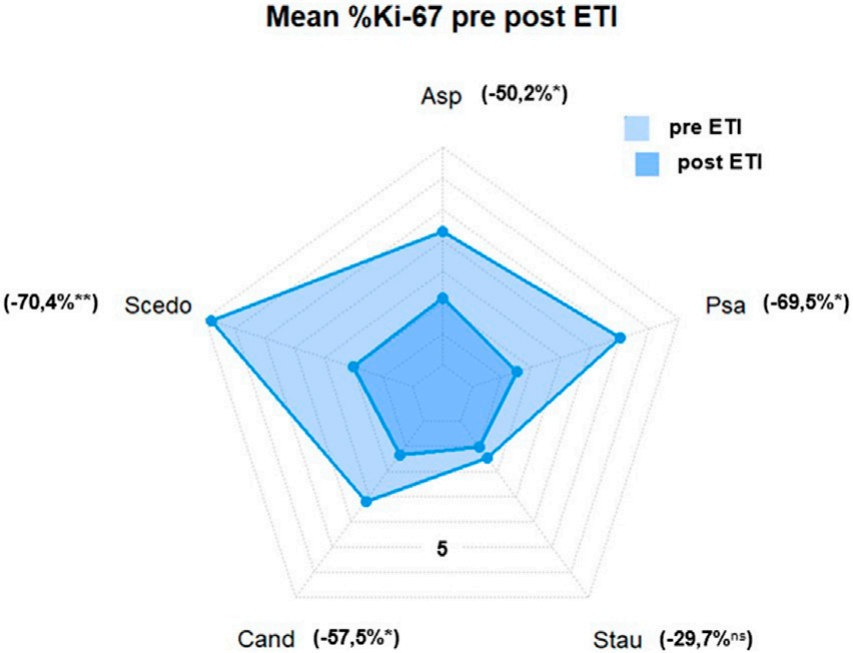
Mean %Ki-67(+) antigen-specific CD154(+) T cells. Asp, aspergillus fumigatus; Scedo, scedosporium apiospermum; Cand, candida albicans; Stau, staphylococcus aureus; Psa, pseudomonas aeruginosa (*) = *p* < 0.05, (**) = *p* < 0.01; ns, not significant.

Mean Ki-67 expression percentage in antigen-reactive CD154 (+)-T cells against *A. fumigatus* (4.28 ± 3.41 vs. 2.13 ± 2.22 (−50.2%), *p* < 0.05), *S. apiospermum* (6.79 ± 4.70 vs. 2.01 ± 2.72 (−70.4%), *p* < 0.01), *C. albicans* (3.18 ± 4.6 vs. 1.35 ± 1.63 (−57.5%), *p* < 0.05), *P. aeruginosa* (5.01 ± 4.10 vs. 1.53 ± 2.67 (−69.5%), *p* < 0.05) decreased significantly after initiation of ETI. We also found a decrease in mean Ki-67 expression percentage in antigen-reactive CD154 (+)-T cells against *S. aureus* (1.45 ± 0.69 vs. 1.02 ± 1.13 (−29.7%), ns), but this difference was not significant (please see Figure 2; Figure 3).

## 4 Discussion

The approval of ETI has improved morbidity and quality of life in pwCF by increasing pulmonary and overall health status (Heijerman et al, 2019; Middleton et al, 2019; Shteinberg and Taylor-Cousar, 2020; Zemanick et al, 2021). It is widely assumed that respiratory disease in pwCF results from a triad of abnormal immune cell responses, pathogen proliferation and proinflammatory respiratory environment (Keown et al, 2020). Therefore, we aimed to examine the impact of ETI *in vivo* on markers of general inflammatory response and of the T-cellular immune response to relevant airway pathogens in relation to their detection in sputum.

In our cohort, only *A. fumigatus* was found significantly less frequently after ETI initiation, which is remarkable, as significantly more patients received long-term antifungal therapy before ETI. In line with this, significantly less exacerbations were experienced, suggesting a reduction of bacterial load. There were no significant qualitative changes for cultural detection of *P. aeruginosa*, *S. aureus*, *S. apiospermum* and *C. albicans*. This partly stands in contrast to recent publications describing reduction of bacterial load and decreased detection of bacterial pathogens in CF respiratory cultures after initiation of ETI (Pallenberg et al, 2022; Beck et al, 2023). Possible causes for this divergence could be the relatively severe phenotype of our cohort and the fact that we were able to examine sputum samples even after ETI initiation. However, due to the improved mucociliary clearance, the samples examined in our study also differed in their quantity before and after ETI, as most patients under ETI were unable to expectorate more than 5 mL of sputum. This may - on the one hand—have had an influence on the bacterial load, which may at least in part explain the lower exacerbation rate. On the other hand, this may have had an influence on the cultural detection rate of *A. fumigatus* after ETI in our study. However, this does not apply to the same extent for the other pathogens we investigated, although—except for *C. albicans*, which was detected more often—the pathogens investigated in our study were also detected slightly less frequently. A link between CFTR dysfunction and defective clearing of *A. fumigatus* is assumed, so CFTR restoration by ETI with more effective immune response may be the main reason for our result (Bercusson et al, 2021). It is also in line with two recent studies, where a rapid significant decrease in *Aspergillus* spp. positive sputum cultures and a restoring of dampened *Aspergillus*-induced reactive oxygen species production by CF phagocytes by CFTR modulators could be shown (Currie et al, 2020; Chesnay et al, 2022).

Interestingly, we found a reduction in the frequencies of Ki-67 positive antigen-specific CD154 (+)-cells for all investigated pathogens independent of their detection rate in sputum samples, which was significant for *P. aeruginosa*, *A. fumigatus*, *S. apiospermum* and *C. albicans.* Significance of Ki-67 decline could not be shown for *S. aureus*, which is most likely due to low Ki-67 frequencies already before ETI initiation in combination with only few numbers of Ki-67 examinations available for this pathogen.

Matching the reduction in Ki-67 frequencies, we found a significant reduction in total serum IgE and total serum IgG. Taken together, these results confirm previous *in vitro* findings of impaired adaptive immune regulation (Mueller et al, 2011; Polverino et al, 2019; Duan et al, 2021) in real life and suggest that CFTR restoration on B and T cells by ETI enables a more effective immune response with a decelerated disease progression (Wheeler et al, 1984). As we found heterogenous changes in microbiology after ETI initiation, reduction of antigen load by improved mucociliary clearance either with or without cross-reactivity between the fungal species examined seems to be only part of the effect of HEMT on lung disease in CF.

Limitations of this study are the relatively small number of patients and that we examined a selected patient cohort with severe phenotype. Nevertheless, these results encourage to be confirmed in a larger cohort of patients and in longitudinal studies to determine whether these effects of HEMT are maintained long-term. Analogous to total IgG, Ki-67 expression on T cells could serve as a general disease severity marker.

## Data Availability

The raw data supporting the conclusion of this article will be made available by the authors, without undue reservation.
